# Catheter ablation combined with left bundle optimized cardiac resynchronization therapy for persistent atrial fibrillation complicated with malignant arrhythmia: A case report

**DOI:** 10.1097/MD.0000000000045047

**Published:** 2025-10-10

**Authors:** Yeshun Wu, Hongqing Xu, Lixia Zhou, Xiaoming Tu, Zhenyan Gao

**Affiliations:** a Department of Cardiology, The Quzhou Affiliated Hospital of Wenzhou Medical University, Quzhou People’s Hospital, Quzhou, China.

**Keywords:** atrial fibrillation, atrioventricular nodal ablation, cardiac resynchronization therapy, case report, malignant arrhythmia

## Abstract

**Rationale:**

Atrial fibrillation (AF) is characterized by rapid and disordered atrial electrical activity. Rhythm and rate control are the primary strategies applied in AF management. In clinical practice, the patient’s symptoms, comorbidities, drug tolerance, cardioversion effectiveness, and risk of disease must be comprehensively evaluated to reasonably recommend strategies for rhythm and rate control.

**Patient concerns:**

A 65-year-old Asian man experienced persistent AF complicated by malignant arrhythmia. He was poorly treated with medications, which led to heart failure and repeated wide QRS complex tachycardia episodes.

**Diagnoses:**

Based on the patient’s overall presentation and electrocardiographic monitoring findings, a diagnosis of persistent AF complicated with malignant arrhythmia and heart failure was made.

**Interventions:**

To restore sinus rhythm, improve myocardial synchronization, and prevent malignant arrhythmia induced by AF, a one-stop treatment strategy of AF catheter ablation combined with atrioventricular nodal ablation and left bundle optimized cardiac resynchronization therapy was adopted for the patient.

**Outcomes:**

At follow-up after 1 year, his N-terminal pro-brain natriuretic peptide level decreased, cardiac shadow reduced, and left ventricular ejection fraction increased. Atrial high-rate episodes were detected without the occurrence of any malignant arrhythmia. Since then, the patient has not developed cardiac insufficiency or syncope.

**Lessons:**

When drug therapy alone is not satisfactorily effective, AF catheter ablation can optimally maintain sinus rhythm. However, considering that sinus rhythm may be difficult to maintain and secondary malignant arrhythmia may be fatal, atrioventricular nodal ablation may be performed to eliminate the effects of AF and rapid ventricular rate, whereas physiological pacing, including left bundle optimized cardiac resynchronization therapy, can be performed to restore the sequence of optimal cardiac contractions. The one-stop comprehensive treatment strategy described herein would be a good option under certain conditions.

## 1. Introduction

Atrial fibrillation (AF), a type of supraventricular arrhythmia, is characterized by rapid and disordered atrial electrical activity. Atrial contraction is affected by disordered electrical activity, and the atrioventricular node exhibits decreasing conduction of rapid atrial excitation. This results in extremely irregular ventricular contractions, leading to decreased cardiac pumping function and heart failure (HF), and even inducing malignant arrhythmias.^[1,2]^ Currently, rhythm and rate control are the main strategies for AF management.^[3–5]^ Rhythm control refers to the administration of antiarrhythmic drugs (AADs), direct current cardioversion, catheter ablation, or surgical ablation for restoring and maintaining sinus rhythm for a long time. Rate control methods include long-term oral medication for controlling the ventricular rate and atrioventricular nodal ablation (AVNA) combined with permanent pacemaker implantation. In clinical practice, it is necessary to comprehensively evaluate the patient’s symptoms, comorbidities, drug tolerance, treatment effectiveness, and risk of disease to reasonably recommend strategies for rhythm and rate control. This article presents the case of a patient with persistent AF complicated with malignant arrhythmia who responded poorly to drug treatments and was treated with AF catheter ablation combined with AVNA and left bundle optimized cardiac resynchronization therapy (LOT-CRT).

## 2. Case description

A 65-year-old Asian man was referred to our clinic in February 2024, with complaints of paroxysmal chest tightness since 3 months and bilateral lower limb edema since 2 weeks. No history of hypertension, diabetes, and cardiovascular and cerebrovascular diseases was reported, and the patients reported to never smoking and drinking.

Three months prior to admission, the patient repeatedly experienced chest tightness when performing physical activities without palpitations and syncope; the symptoms were relieved after a short resting period. He was admitted to the local hospital, where coronary angiography revealed mild coronary stenosis without stent implantation, Holter electrocardiogram (ECG) indicated rapid AF, echocardiogram revealed left heart and right atrial enlargement and a left ventricular ejection fraction (LVEF) of 41.3%, and N-terminal-pro brain natriuretic peptide (NT-proBNP) level of 1900 ng/L. His symptoms were alleviated after treatment with metoprolol (47.5 mg, qd), valsartan/sacubitril (50 mg, bid), furosemide (20 mg, qd), spironolactone (20 mg, bid), empagliflozin (10 mg, qd), rivaroxaban (20 mg, qd), and digoxin (0.125 mg, qd), and he was discharged. However, 1 month ago, he stopped taking the medications without medical consultation. Two weeks ago, the patient reeexperienced chest tightness during physical activity and gradually developed bilateral lower limb edema. He was readmitted to the local hospital, and reexamination revealed an NT-proBNP level of 12,700 ng/L. During hospitalization, he developed sudden sweating episodes and fatigue. Real-time ECG monitoring indicated a heart rate of > 200 beats/min (bpm), followed by urinary and fecal incontinence despite clear consciousness. The patient’s condition improved after appropriate treatment (the medical records not obtained from the local hospital), and he was immediately transferred to our hospital, with the diagnosis of arrhythmia-induced cardiomyopathy, persistent AF, and HF. His vital signs upon presentation were as follows: blood pressure, 116/89 mm Hg; irregular heart rate, 80 bpm; and oxygen saturation, 98%. Physical examination revealed mild bilateral lower limb edema, and auscultation revealed slight moist crackles in both lungs but no pathological murmurs in the heart. On echocardiogram, the left atrial anteroposterior diameter (LAAPD) was 4.9 cm, left ventricular internal diameter at end-diastole (LVIDd) was 6.4 cm, and LVEF was 37%.

**Figure 1.**
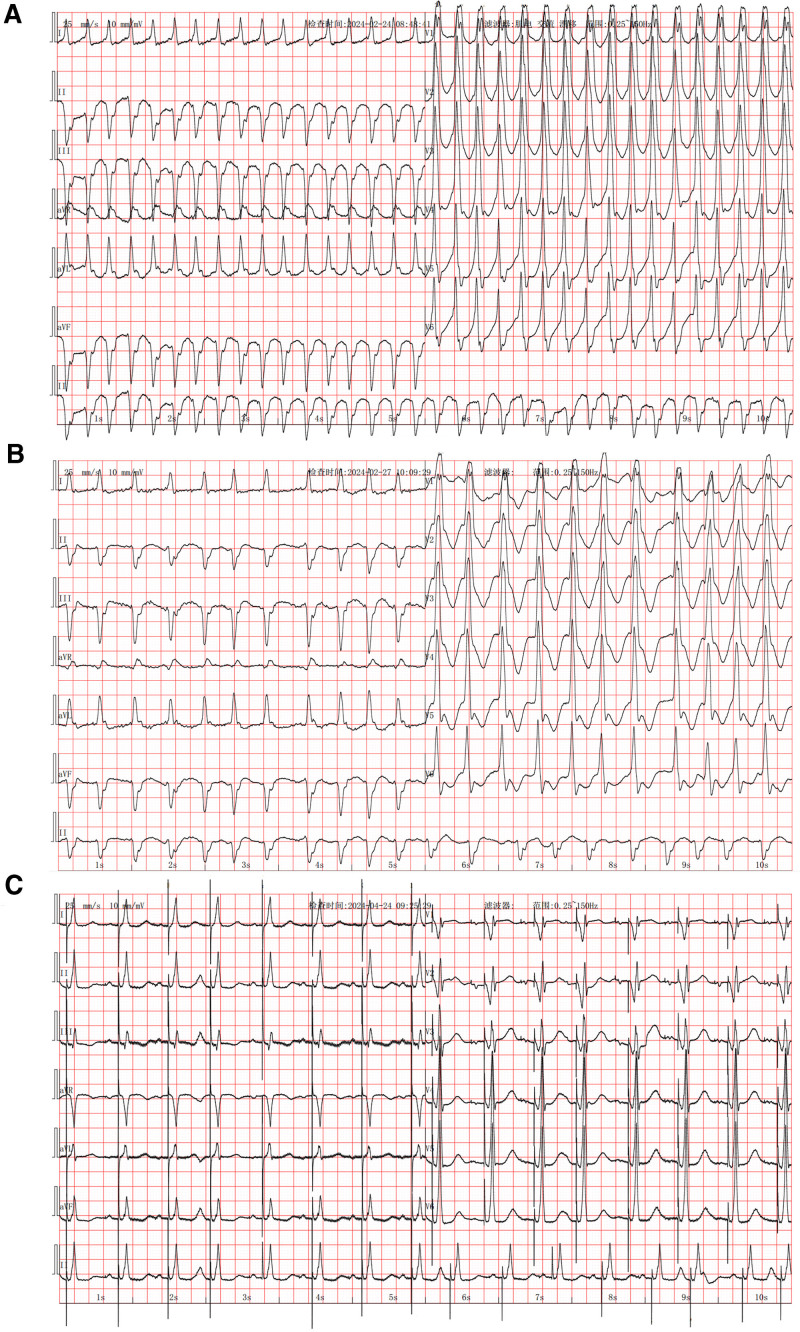
(A) ECG showing wide QRS complex tachycardia. (B) ECG showing rapid AF combined with complete right bundle branch block and left anterior branch block. (C) ECG showing sinus rhythm and atrioventricular sequential pacing, with DDD and VAT pacing. AF = atrial fibrillation, DDD = dual chamber demand, ECG = electrocardiogram, VAT = atrial-triggered ventricular.

**Figure 2.**
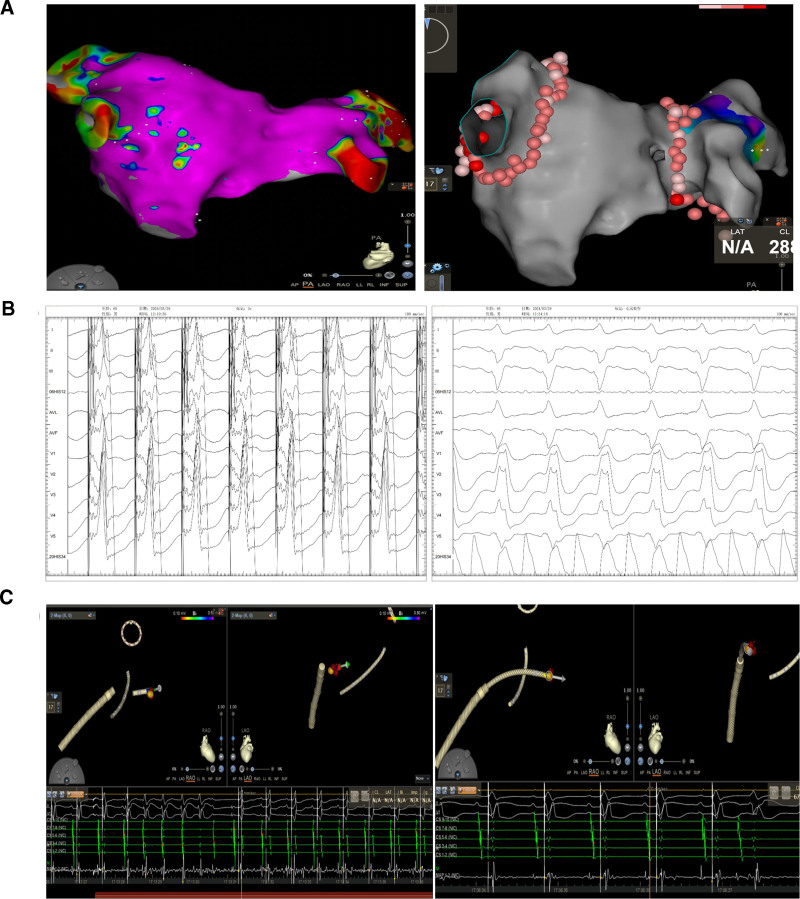
(A) Catheter-based circumferential pulmonary vein isolation. Three-dimensional mapping of the left atrium and pulmonary vein generated using the CARTO3 system (Biosense Webster, Diamond Bar), and catheter ablation of the left and right pulmonary vein vestibules to convert AF into sinus rhythm. Test by Lasso electrode confirming that the potentials of the left upper and lower pulmonary veins and the right upper and lower pulmonary veins disappeared, and both afferent and efferent currents of the bilateral pulmonary veins are blocked. (B) Intracardiac electrogram during left bundle branch area pacing and low atrial septal pacing. After routine skin disinfection, the pacing system is implanted via the left axillary vein. Right ventricular and atrial leads are implanted in the right ventricular septum and the low atrial septum, respectively. A 3830 electrode lead (Medtronic Incorporation, Minneapolis) is inserted into the tricuspid annulus through the C315HIS catheter (Medtronic Incorporation) and is rotated until the pacing rhythm shows the right bundle branch block. Next, another 3830 electrode lead is inserted into the right atrium through the C315 S4 catheter (Medtronic Incorporation). Fluoroscopy is performed at 35° in the left anterior oblique position, and the catheter and lead tip are adjusted to point in the direction of the spine before electrode insertion. Finally, electrode parameters are optimized, electrode tension is adjusted, and electrodes are fixed. The pacemaker is connected to the atrial and ventricular electrodes, placed in a pouch, and the skin is sutured layer by layer. (C) Atrioventricular nodal ablation. The atrioventricular node area is measured using the 3D mapping, and the HIS bundle potential is detected. Discharging with a temperature control of 43°C and a power of 35 W, and irrigating with cold saline at 17 mL/min. After 4 s, a whole ventricular pacing appeared, indicating complete atrioventricular block. Ablation is continued for 2 min, and whole ventricular pacing is observed at different frequencies of atrial pacing, considering complete atrioventricular node block.

On the day after admission, he experienced sudden hot flashes and chest tightness; his blood pressure was 74/49 mm Hg and ECG revealed wide QRS complex tachycardia (Fig. 1A). Diazepam was administered for sedation, followed by electrical cardioversion; subsequently, the rhythm was converted to AF with a heart rate of 85 to 120 bpm. To prevent tachycardia, esmolol was sustainably administered. However, 3 days later, the patient developed sudden limb convulsions and loss of consciousness, and real-time ECG monitoring showed ventricular asystole. Cardiopulmonary resuscitation was immediately performed. After 2 minutes, real-time ECG monitoring revealed wide QRS complex tachycardia that got converted to sinus rhythm following electrical cardioversion, with intermittent complete atrioventricular block. A temporary pacemaker was implanted. Tachycardia recurred within 10 minutes, and repeat ECG indicated rapid AF combined with complete right bundle branch block and left anterior branch block (Fig. 1B). Following medication, the patient experienced recurrent malignant rapid and slow arrhythmias. After discussing within the department and obtaining the patient’s consent, AF catheter ablation was performed in combination with AVNA and LOT-CRT (Fig. 2).

**Figure 3.**
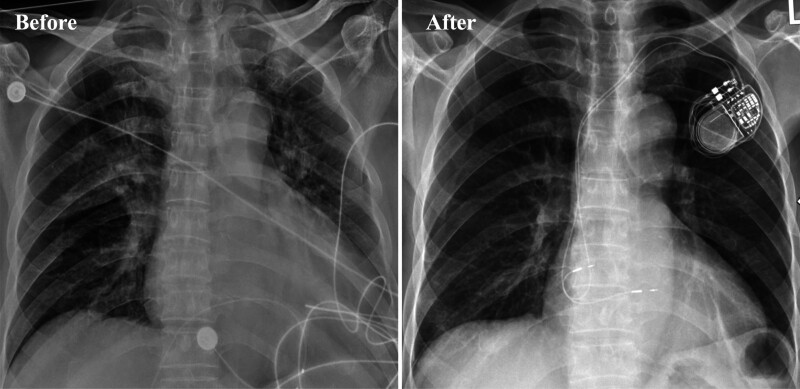
Chest X-ray before operation and at 1-yr follow-up.

**Figure 4.**
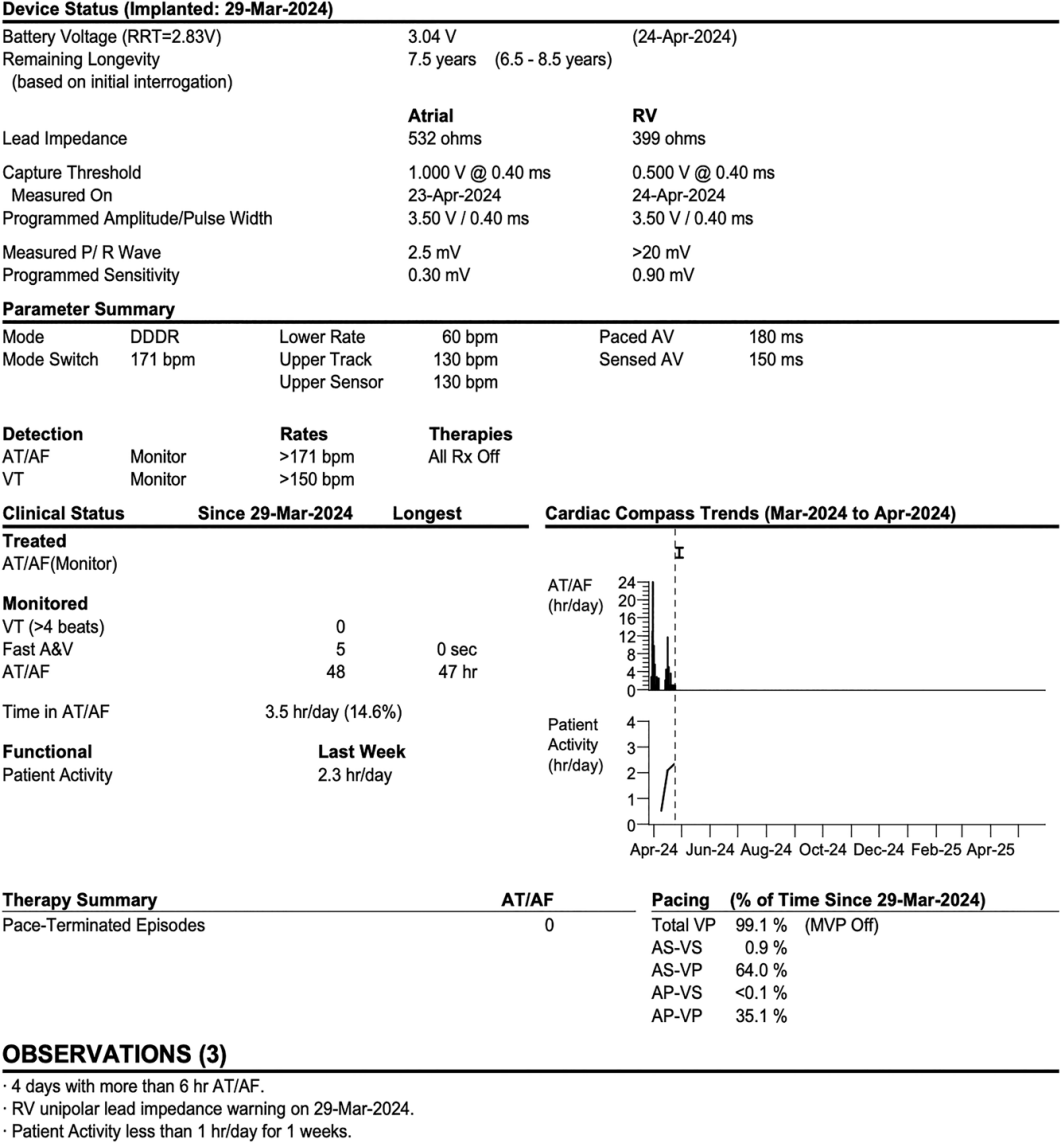
Pacemaker parameters at 4-wk follow-up.

**Figure 5.**
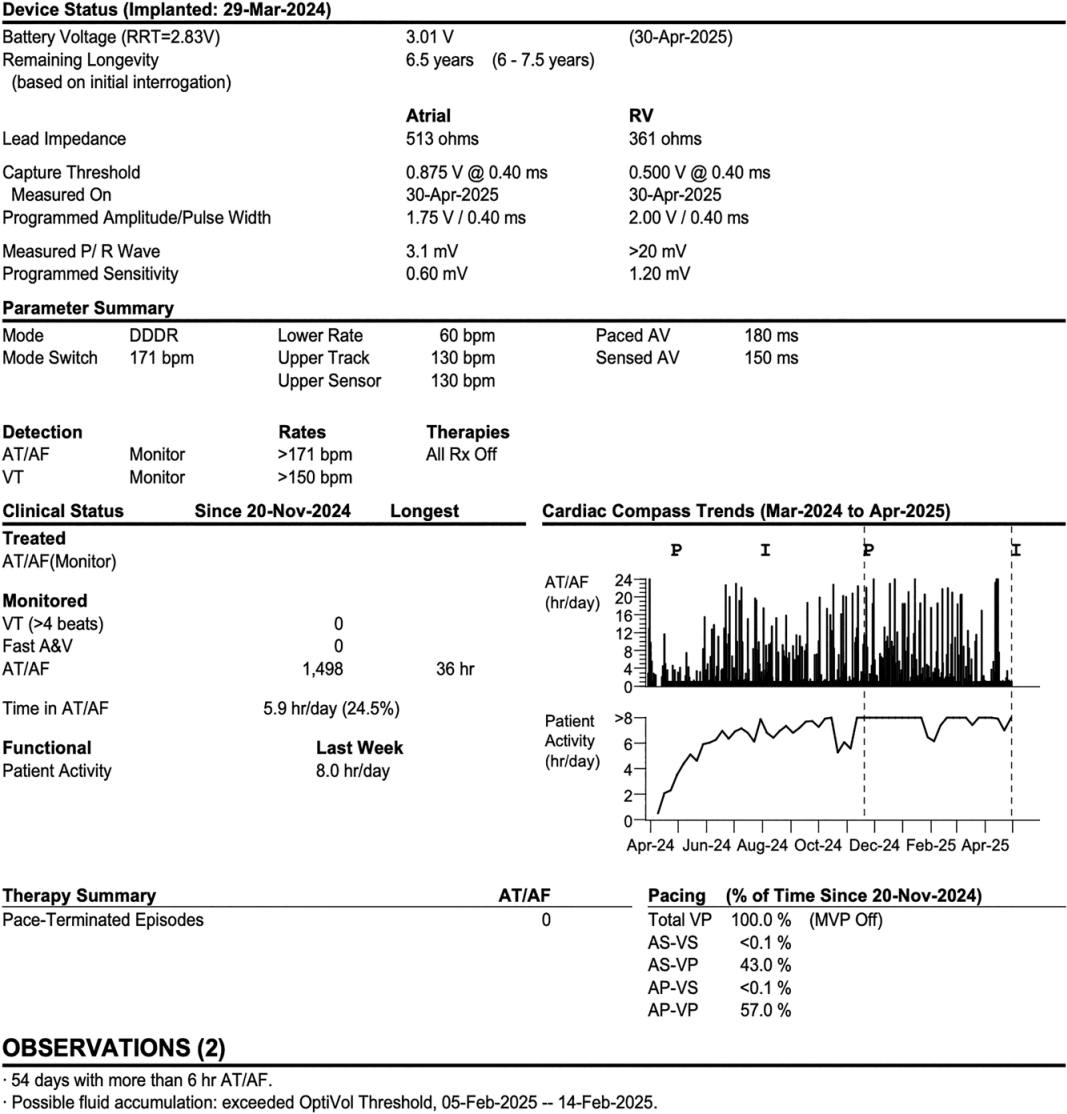
Pacemaker parameters at 1-yr follow-up.

**Figure 6.**
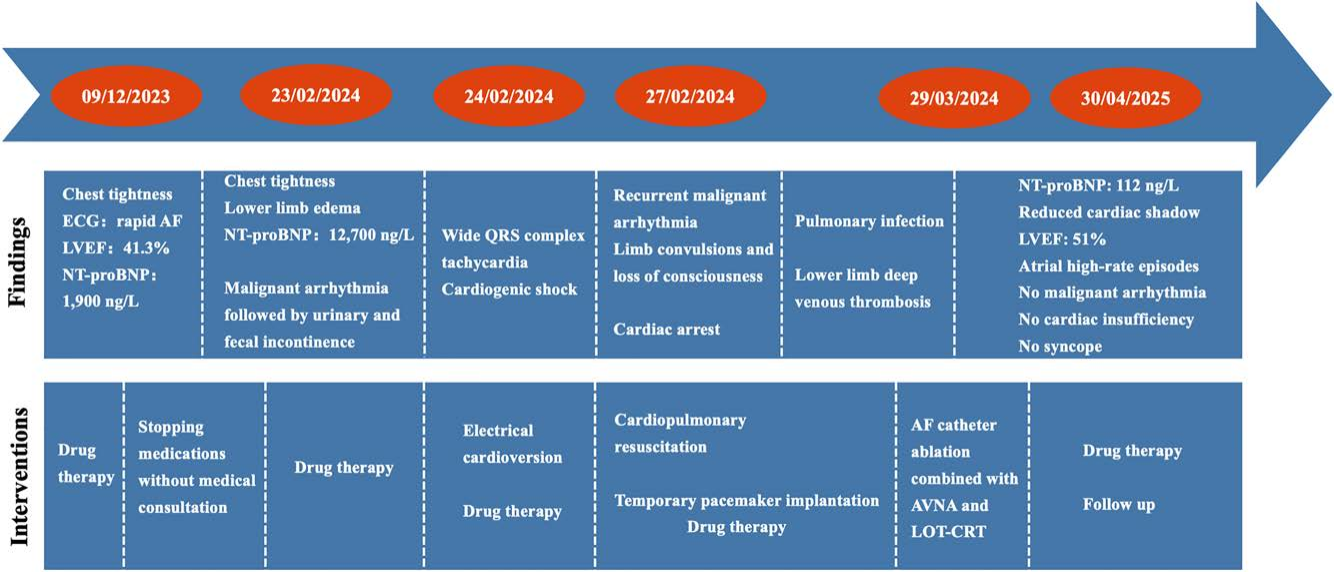
Case progress timeline. AF = atrial fibrillation, AVNA = atrioventricular nodal ablation, ECG = electrocardiogram, LOT-CRT = left bundle optimized cardiac resynchronization therapy, LVEF = left ventricular ejection fraction, NT-proBNP = N-terminal-pro brain natriuretic peptide.

During the postoperative hospitalization period (7 days), no tachycardia recurrence was observed. The patient’s condition improved, and he was discharged with the following prescribed medications: furosemide (20 mg, qd), spironolactone (20 mg, bid), rivaroxaban (20 mg, qd), dapagliflozin (10 mg, qd), metoprolol (23.75 mg, qd), amiodarone (200 mg, bid), and trimetazidine (20 mg, tid). At follow-up after 4 weeks, his NT-proBNP level was 460 ng/L, and ECG indicated sinus rhythm and atrioventricular sequential pacing with dual chamber demand (DDD) and atrial-triggered ventricular (VAT) pacing (Fig. 1C). At follow-up after 1 year, his NT-proBNP level was 112 ng/L, and chest X-ray revealed a significantly reduced cardiac shadow (Fig. 3). The echocardiogram revealed LAAPD, LVIDd, and LVEF of 4.2 cm, 5.9 cm, and 51%, respectively. The pacemaker parameters remained stable (Figs. 4 and 5), whereas atrial high-rate episodes were detected without any malignant arrhythmia occurrence. The patient has since not developed cardiac insufficiency or syncope (Fig. 6).

## 3. Discussion

The patient had rapid persistent AF, which was poorly treated with medications at a previous hospital, thereby leading to HF and repeated episodes of wide QRS complex tachycardia. Moreover, the patient presented with intermittent complete atrioventricular block and Adams–Strokes syndrome following AADs administration. Therefore, radiofrequency ablation and cardiac resynchronization therapy (CRT) were more likely to improve the long-term prognosis of the patient than drug therapy.

AF and HF share common risk factors and are causal to each other.^[6,7]^ HF triggers and maintains AF by causing neuroendocrine disorders, abnormal cardiac electrical activity, and cardiac remodeling. AF leading to loss of atrial systolic function, increased ventricular rate, and irregular ventricular movement, which then exacerbates HF. The RE-LY trial reported HF as the leading cause of death within 1 year for patients who presented to a hospital emergency department with AF.^[8]^ Another meta-analysis involving 18 randomized controlled trials indicated that all-cause mortality in patients with AF is significantly reduced by catheter ablation, primarily because of its significant benefits in patients with AF and HF with reduced ejection fraction.^[9]^ Therefore, for treating AF combined with HF, blocking the interactive and vicious cycle between the 2 is extremely important, and the selected treatment strategy should comprehensively consider the effective AF control and HF prevention.

Rhythm and rate control are the 2 main strategies for AF treatment. Catheter ablation for rhythm control has accumulated sufficient evidence and can be used to effectively reduce the risk of AF recurrence and cardiovascular hospitalization compared with ADD.^[9–11]^ An observational study involving 183,760 patients with AF who underwent catheter ablation or drug therapy during the same period proposed that catheter ablation is significantly associated with a reduced composite endpoint of all-cause death, stroke, major bleeding, and cardiac arrest.^[10]^ Compared to medical therapy, catheter ablation therapy for AF in HF patients is associated with greater improvements in LVEF and quality of life.^[12]^ Furthermore, in patients with AF and end-stage HF, compared with drug therapy alone, the combination of catheter ablation and guideline-guided drug therapy significantly reduces the likelihood of all-cause death, left ventricular assist device implantation, or emergency heart transplantation.^[13]^ Additionally, restoring sinus rhythm can reverse the increase in atrioventricular volume, alleviate functional valve regurgitation, and subsequently improve cardiac remodeling.^[14]^

However, in some patients with AF, maintaining sinus rhythm following catheter ablation is relatively difficult, despite having a durable pulmonary vein isolation.^[15]^ All major guidelines for AF management have suggested that AVNA combined with CRT should be considered (Class IIa, Level of evidence B) for patients with severe symptoms, inadequate control of the ventricular rate by medication, and difficulty in maintaining sinus rhythm through catheter ablation.^[3–5]^ Recently, left bundle branch area pacing (LBBAP) has been an acceptable approach for physiological pacing, and LOT-CRT has proven effective and advantageous in achieving cardiac synchronization.^[16–18]^ An international clinical study involving 15 centers showed that in patients with HF and CRT indications, right ventricular electrode implantation into the left bundle branch area can effectively shorten the pacing QRS duration. Compared with traditional biventricular pacing, LBBAP can prolong the time to death or HF hospitalization and improve clinical outcomes in patients with CRT indications.^[19]^ Moreover, in patients with HF and AF and in those with implantable cardioverter defibrillator in whom ventricular rates are difficult to be controlled by medications, AVNA combined with LOT-CRT has been confirmed as feasible, safe, and effective with a high success rate. In addition, it reduces the use of oral drugs and decreases mortality and hospitalization risks.^[20–22]^ Briefly, the following are the advantages of AVNA combined with LOT-CRT: it can block the transmission of AF to the ventricle, thereby achieving the goal of rate and rhythm control; it can improve cardiac remodeling and prevent HF, leading to more significant improvement in patients’ clinical symptoms; and it can reduce the need for AADs administration and the associated side effects.

In this case, considering that the patient experienced poor efficacy of AADs, drug side effects, and may have difficulty in maintaining sinus rhythm after catheter ablation, a one-stop treatment strategy of AF catheter ablation combined with AVNA and LOT-CRT was adopted to restore sinus rhythm as long as possible, improve myocardial synchronization, and prevent malignant arrhythmia induced by potentially recrudescent AF. Based on the recurring AF, the relief in clinical symptoms, decreased NT-proBNP levels, reduced cardiac shadow, and increased LVEF during 1-year follow-up, it is suggested that AF catheter ablation combined with AVNA and LOT-CRT has a relatively ideal application prospect for this type of cases. However, prospective large-scale randomized clinical trials are warranted to confirm long-term effectiveness and safety.

## 4. Conclusion

Rate and rhythm control are equally significant in patients with rapid AF and HF. When drug therapy alone is not satisfactorily effective, AF catheter ablation can optimally maintain sinus rhythm. However, considering that sinus rhythm may be difficult to maintain and secondary malignant arrhythmia induced by AF may be fatal, AVNA may be performed to eliminate the effects of AF and rapid ventricular rate, while physiological pacing, including LOT-CRT, can be performed to restore the sequence of optimal cardiac contractions. Under certain conditions, the current performed one-stop comprehensive treatment strategy would be a good option.

## Acknowledgments

We thank all the medical staff members who were involved in treating the patient.

## Author contributions

**Conceptualization:** Zhenyan Gao.

**Data curation:** Yeshun Wu, Hongqing Xu.

**Investigation:** Yeshun Wu, Hongqing Xu, Lixia Zhou, Xiaoming Tu.

**Supervision:** Xiaoming Tu, Zhenyan Gao.

**Writing – original draft:** Yeshun Wu.

**Writing – review & editing:** Zhenyan Gao.
